# Differential Effects of *Naja naja atra* Venom on Immune Activity

**DOI:** 10.1155/2014/287631

**Published:** 2014-06-12

**Authors:** Jian-Qun Kou, Rong Han, Yin-Li Xu, Xiao-Lan Ding, Shu-Zhi Wang, Cao-Xin Chen, Hong-Zhang Ji, Zhi-Hui Ding, Zheng-Hong Qin

**Affiliations:** ^1^Jiangsu Key Laboratory of Translational Research and Therapy for Neuro-Psycho-Diseases (BM2013003), Department of Pharmacology and Laboratory of Aging and Nervous Diseases, Soochow University School of Pharmaceutical Science, Suzhou 215123, China; ^2^The First Affiliated Hospital of Soochow University, Suzhou 215006, China

## Abstract

Previous studies reported that *Naja naja atra* venom (NNAV) inhibited inflammation and adjuvant arthritis. Here we investigated the role of NNAV in regulation of immune responses in mice. Oral administration of NNAV to normal mice showed significant increase in natural killer cell activity, B lymphocyte proliferation stimulated by lipopolysaccharides, and antibody production in response to sheep red blood cells. Meanwhile, NNAV markedly decreased T lymphocyte proliferation stimulated by concanavalin A, arrested the cell cycle at G_0_/G_1_ phase, and suppressed CD4 and CD8 T cell divisions. Furthermore, NNAV inhibited the dinitrofluorobenzene-induced delayed-type hypersensitivity reaction. This modulation of immune responses may be partly attributed to the selective increase in Th1 and Th2 cytokines (IFN-*γ*, IL-4) secretion and inhibition of Th17 cytokine (IL-17) production. In dexamethasone-induced immunosuppressed mice, NNAV restored the concentration of serum IgG and IgM, while decreasing the percentage of CD4 and CD8 T-cell subsets. These results indicate that NNAV enhances the innate and humoral immune responses while inhibiting CD4 Th17 and CD8 T cell actions, suggesting that NNAV could be a potential therapeutic agent for autoimmune diseases.

## 1. Introduction

Immune system is a defense mechanism for protecting individuals from being attacked by foreign organisms or abnormal cells. The immune system will be activated in response to a threat from a foreign organism and when the threat has been removed it has to return to its basal level. While the immune system can fail, giving rise to immunodeficiency, leading to overwhelming infection, it can, on the other hand, overreact against foreign organisms leading to tissue damage. If immune system overreacts against the body's own cells, it leads to autoimmune diseases. It is essential that the immune system be tightly regulated.

Snake venom is composed of a number of enzymes, peptides, and proteins with different biological functions. NNAV contains cobra venom factor (CVF), cardiotoxin (CTX), cobratoxin (or cobrotoxin), phospholipases A2 (PLA2), and many other components. There were a variety of reports showing that the snake venom from* Naja naja atra* and its components had analgesic and anti-inflammatory effects [1, 2]. CVF is an anticomplement protein and has been used for preventing immune rejection in experimental organ transplantation [3–5]. CTX-3 has been reported to have an antitumor property [6]. It was also revealed that CTX-3 and PLA2 had bactericidal activity and would be a new tool against bacterial diseases [7, 8]. Our previous studies showed that cobratoxin had anti-inflammatory effects on rheumatoid arthritis (RA), a systemic autoimmune disease [9]. NF-*κ*B is an important target for the inhibition of T-cell proliferation and activation [10]. Our previous studies showed that cobrotoxin had an inhibitory effect on activation of NF-*κ*B [11]. However, the systemic study of NNAV on immune system has not been explored.

There is an assumption that oral administration of peptides may lead to inactivation of peptides caused by the chemical degradation or enzymatic digestion in the gastrointestinal tract. However, several studies showed that, when given orally, either a low molecular weight component from* Crotalus terrificus* venom [12] or neurotoxin from* king cobra* [13] produced analgesic effects. It was also confirmed the absorption of ^125^I-labeled neurotoxin of NNAV from the rectum of rabbits [14]. Cobra venoms are characterized by their toxic activity; removal of the toxicity of the venom can be achieved by exposure to heat without influence on the immunogenicity [15, 16]. In our previous studies we reported protective effects of NNAV on RA and nephropathy by oral administration [17, 18], suggesting that NNAV can be absorbed to produce pharmacological effects. The objective of this study was to evaluate the influence of the heat-treated NNAV on innate, humoral, and cellular immune responses in normal and dexamethasone-induced immunosuppressed mice.

## 2. Materials and Methods

### 2.1. Animals

The ICR mice were used to study the effects of NNAV on immune activity in healthy mice, and the C57BL/6J mice were used to study the effects of NNAV on immune activity in dexamethasone-induced immunosuppressed mice. All mice, weighing 18~20 g, were provided by the Shanghai Slac Laboratory Animal Co., Ltd. Food and tap water were available ad libitum. The animal room was maintained on a 12 h light/dark cycle with constant temperature of 23 ± 2°C and 50 ± 5% humidity. The experimental procedures were approved by the animal care and use committee of Soochow University and were conducted based on the National Institutes of Health guidelines for the Care and Use of Laboratory Animals (NIH Publications No. 80-23, revised 1996). And all efforts were made to minimize the suffering of mice.

### 2.2. Drug Administration

The NNAV lyophilized powder was purchased from the Rainbow Snake Farm (Yu Jiang, Jiangxi Province, China) and was dissolved in distilled water, heated in boiling water for 10 minutes, and slowly cooled down to room temperature [17]. NNAV at doses of 20, 40, and 80 *μ*g/kg was administered by p.o. gavage to mice once a day for 21 consecutive days. Control animals received water at the same volume.

### 2.3. Determination of Body Weight and Lymphoid Organ Index

The body weights of the mice from each group were measured at the end of study. The thymus and spleen samples were collected and weighted immediately after mice were sacrificed. The lymphoid organ index was calculated as follows: lymphoid organ index (mg/g) = lymphoid organ weight/body weight ×  10.

### 2.4. Splenocyte Preparation

Splenocyte suspensions were prepared as previously described with minor modifications [19]. In brief, after 21 days of drug treatment, spleens were removed aseptically, cut into small pieces, and then passed through a tissue sieve (200 meshes per 2.5 cm, Shanghai Solarbio Science & Technology Co., Ltd., Shanghai, China) in 2 mL PBS to remove major tissue aggregates. Cell suspensions from spleens were centrifuged at 1500 r/min for 5 min, and then resuspended in 150 mM NH_4_Cl, 10 mM KHCO_3_, and 0.1 mM EDTA for 5 min to lyse red cells. Cells were washed two times and finally suspended in 2 mL RPMI-1640 medium (Gibco, Gaithersburg, MD, USA) with 10% fetal bovine serum (FBS; Gibco, Gaithersburg, MD, USA). The viability of cells in suspension was about 95% according to the trypan blue dye (Shanghai Solarbio Science & Technology Co., Ltd., Shanghai, China) exclusion test, and cells were counted before being plated into culture dishes.

### 2.5. Flow Cytometric Analysis of T Lymphocyte Subpopulations

The percentages of T lymphocyte subpopulations from spleen were measured with flow cytometric analysis [20]. Splenocyte suspensions (1 × 10^6^ cells/mL) were stained with anti-mouse CD3e-PE-cy5, CD4-PE, and CD8a-FITC antibodies (eBioscience, San Diego, CA, USA) for 20 min at room temperature in the dark. Then cells were washed with PBS to remove the excess stains. Each sample was suspended in 500 *μ*L of PBS and analyzed with a flow cytometer (FC500, Beckman Counter, USA). The data were acquired by gating on CD3^+^CD4^+^ or CD3^+^CD8^+^ cell population.

### 2.6. NK Cell Activity

Natural killer (NK) cell activity of spleen cells was determined with a short-term lactate dehydrogenase (LDH) release assay [21]. Complement of the FBS was inactivated at 56°C for 30 min before assay. Both effector cells (NK cells) and target cells, a cell line of Moloney virus induced lymphoma of A/Sn organ and with noted sensitivity to NK cells (YAC-1 cells, Shanghai Institute of Biochemistry and Cell Biology, Shanghai, China), were washed two times in RPMI-1640 with 5% FBS. The effector cells were incubated for 4 h with target cells at 10 : 1 ratio, that is, 100 *μ*L effector cells (5 × 10^6^ cells/mL) and 100 *μ*L target cells (5 × 10^5^ cells/mL), followed by centrifugation at 1800 r/min for 5 min. LDH activity in the cell culture supernatants was measured using the LDH release cytotoxicity detection kit (Beyotime Institute of Biotechnology, Nantong, China) and quantitated by measuring absorbance at 490 nm using a plate reader (SpectraMax M5, USA). The release of LDH upon lyses of YAC-1 cells was regarded as the maximum (100%), and the LDH release from YAC-1 cells alone was served as the spontaneous baseline.

### 2.7. Humoral Antibody Response to SRBC

Sheep red blood cells (SRBCs; Guangzhou Ruite Bio-tec Co., Ltd., Guangzhou, China) were kept in Alsever's medium and washed three times with normal saline before use and centrifuged at 2000 rpm for 5 min. Mice of model and NNAV-treated group were sensitized with 5% SRBC (10^9^ cells/mL) 0.2 mL per mouse by i.v. injection on day 16th of NNAV administration; normal group received the same volume of saline. Five days later blood samples were collected from retroorbital plexuses. The amount of circulating anti-SRBC antibody was determined by examining the capacity of 1 : 300 dilution of test serum to lyse SRBC in the presence of guinea pig complement (Guangzhou Ruite Bio-tec Co., Ltd., Guangzhou, China). Briefly, 250 *μ*L of 2.5% SRBC was added to 100 *μ*L diluted serum, followed by 250 *μ*L 10% guinea pig complement in sequence. All the samples were incubated for 1 h at 37°C and then centrifuged for 5 min at 1800 rpm. The amount of lysed SRBC in supernatant was collected and detected using a plate reader (SpectraMax M5, USA) at 540 nm [22]. The higher concentration of anti-SRBC antibody will result in the lysis of larger number of SRBC and thus generate greater OD values in the supernatant.

### 2.8. Lymphocyte Proliferation Assay

The cell proliferation was determined with cell counting kit-8 (CCK-8) [23]. Briefly, 100 *μ*L of spleen cell suspension (5 × 10^6^ cells/mL) from each mouse was added into individual wells of 96-well plates. The splenocytes were cultured for 48 h in RPMI 1640 complete medium and stimulated with or without T-cell mitogen concanavalin A (ConA; 5 *μ*g/mL final concentration; Sigma, St. Louis, Missouri, USA) or B-cell mitogen lipopolysaccharide (LPS; 10 *μ*g/mL final concentration; Sigma, St. Louis, Missouri, USA) to make a final volume of 200 *μ*L. After 44 h, 20 *μ*L of CCK-8 solution from a nonradioactive cell counting kit (Dojido, Kumamoto, Japan) was added to each well. Then plates were incubated for 4 h. The absorbance of each sample was read at 450 nm using a plate reader (SpectraMax M5, USA). The T or B-cell proliferation rate was calculated as follows: proliferation rate % = (OD_stimulated  cells_ − OD_non-stimulated  cells_)/OD_non-stimulate  cells_ × 100%.

### 2.9. T-Cell Division Analysis

To examine the effects of NNAV on cell division, splenocytes were stained with the 5-carboxyfluorescein diacetate succinimide ester (CFSE; eBioscience, San Diego, CA, USA) at the concentration of 2.5 *μ*M and then incubated for 10 min at room temperature in the dark. The staining was stopped by adding 5 volumes of cold complete media and incubated on ice for 5 min. Labeled cells were washed 3 times with complete media and then counted, plated, and stimulated with 5 *μ*g/mL ConA for 72 h. Cells were collected and incubated with anti-mouse CD4-PE or CD8a-PE antibody (eBioscience, San Diego, CA, USA). The data were acquired by gating on CD4^+^ or CD8^+^ cell population on flow cytometer (FC500, Beckman Counter, USA). The sequential loss of CFSE fluorescence was used to determine the cell division [24].

### 2.10. T-Cell Cycle Progression Analysis

Splenocytes (2 × 10^5^ cells/well) were seeded onto 96-well plate and treated with 5 *μ*g/mL ConA for 48 h. Cells were then harvested, washed, and fixed in 70% ice-cooled ethanol at 4°C for 24 h. Then cells were centrifuged and washed with cooled PBS. Cells were suspended in 500 *μ*L PBS and stained with 20 *μ*L propidium iodide (PI; 1 mg/mL; Beyotime Institute of Biotechnology, Nantong, China) containing 20 *μ*L Rnase A (20 mg/mL; Beyotime Institute of Biotechnology, Nantong, China) for 1 h at 37°C. The cell cycle phase was determined using flow cytometer (FC500, Beckman Counter, USA). The percentage of cells in G_0_/G_1_, S and G_2_/M phases was calculated with Multicycle software. The sub-G_1_ peak in the cell cycle was quantified to measure the apoptotic cells [25].

### 2.11. ELISA Assay

For cytokines assay [26], splenocytes were cultured as described in the proliferation assay and stimulated with ConA (5 *μ*g/mL). Supernatants were harvested by centrifugation after 48 h of incubation. The concentrations of IFN-*γ*, IL-4, and IL-17 were assayed with ELISA kits (Shanghai Hushang Biotechnology Co., Ltd., Shanghai, China or eBioscience, San Diego, CA, USA).

### 2.12. Intracellular Cytokine Staining

Intracellular cytokine staining was performed as previously described [16]. In brief, 2.5 × 10^6^ cells from control and NNAV-treated mice were stimulated with Phorbol-12-myristate-13-acetate (PMA, 50 ng/mL final concentration; Sigma, St. Louis, Missouri, USA) plus ionomycin (1 *μ*M; eBioscience, San Diego, CA, USA) and brefeldin A (BFA; 1 : 1000 dilution, eBioscience, San Diego, CA, USA) for 6 h in 24-well plate. After stimulation, cells were collected and stained with anti-mouse CD3e-APC and CD4-PE antibody (eBioscience, San Diego, CA, USA) for 20 min at room temperature. Cells were washed and fixed with fixation buffer (eBioscience, San Diego, CA, USA) for 25 min at room temperature. Fixed cells were washed twice with permeabilization buffer (eBioscience, San Diego, CA, USA) and then intracellular cytokine staining was carried out using FITC conjugated anti-mouse IFN-*γ*, IL-4, or IL-17 antibody (eBioscience, San Diego, CA, USA) for 20 min. Samples were analyzed with BD FACScan (BD Biosciences, USA) using Cell Quest software (BD Biosciences, USA).

### 2.13. Delayed-Type Hypersensitivity

On the 15th day of NNAV administration, model and NNAV-treated mice were immunized by painting 100 *μ*L of 0.5% dinitrofluorobenzene (DNFB; Shanghai Baomanbio Co., Ltd., Shanghai, China) in acetone : olive oil (4 : 1) on the shaved back, and normal mice were immunized by painting with 100 *μ*L vehicles alone. Five days later, 20 *μ*L of 0.2% DNFB was applied on the right ear, and the vehicle was used on the left ear as a control. Animals were sacrificed 24 h after challenge. The ears were rapidly removed and an 8 mm diameter punch in the central part of each ear was made by a biopsy punch (YLS-Q4, Jinan Yiyan Co., Ltd., Jinan, China). The punches were weighted immediately. The swelling rate was calculated as follows: swelling rate % = (right ear wt. − left ear wt.)/left ear wt. × 100% [27].

### 2.14. Dexamethasone-Induced Immunosuppression

The C57BL/6J mice were immunosuppressed by oral administration of dexamethasone (DEX; Hubei Tianyao Pharmaceutical Co., Ltd., Hubei, China) at the dose of 1 mg/kg daily for 7 days [28], and then NNAV was administered in the following 21 days. Mice continued to receive DEX at the dose of 0.25 mg/kg to maintain adequate immunosuppression throughout the duration of the NNAV treatment [29]. Twenty-one days after NNAV administration, blood samples were collected from retroorbital plexuses and the serum was separated for the determination of IgG and IgM using ELISA kits (eBioscience, San Diego, CA, USA). Spleens were collected for the determination of T lymphocyte subsets with flow cytometer as previously described or fixed in 10% PBS-buffered formalin and embedded in paraffin for histological assessment of the lesions with hematoxylin and eosin (H&E) staining.

### 2.15. Statistical Analysis

All results were expressed as mean ± standard deviation (SD). Data were statistically analyzed for differences with one-way analysis of variance (ANOVA) with an appropriate* post hoc* test or unpaired* t*-test using SPSS 16.0 software. Statistical significance was accepted at *P* < 0.05.

## 3. Results

### 3.1. NNAV Increased Innate and Humoral Immune Activity

During the course of study, we observed if oral administration of NNAV induced toxic responses. According to our observation, NNAV did not result in any mortality or abnormal behaviors during the experiment. As shown in Table 1, NNAV had no influence on body weight, lymphoid organ index, or T lymphocyte differential count. These indices suggest that NNAV did not produce apparent toxic effect in mice at the doses used in the study.

**Figure 1:**
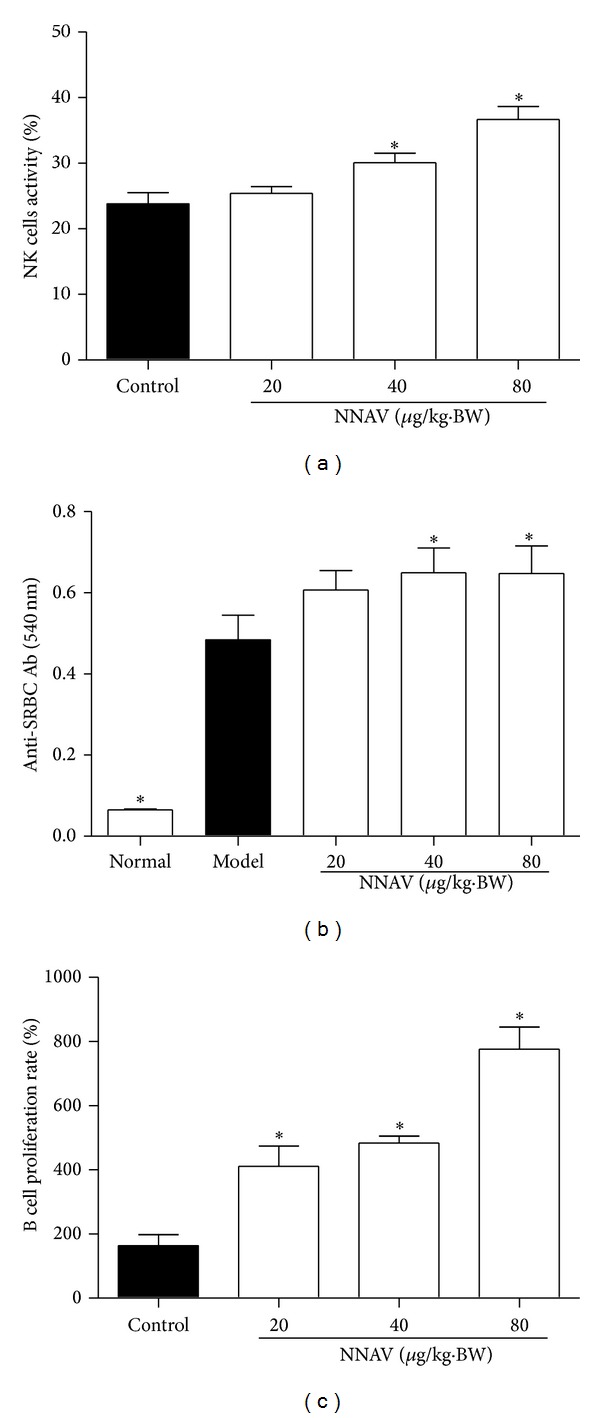
NNAV increased innate and humoral immune activity. NNAV (20~80 *μ*g/kg) was administered orally to ICR mice for 21 days, and control mice were given water instead. (a) The effector cells isolated from the spleens of NNAV-treated or control mice were cultured with target cells (YAC-1 cell line) for 4 h at the ratio of 10 : 1. The NK cell activity was determined using the LDH release assay. Values represent mean values ± SD from 4 mice per group; triplicates were used for each mouse.   *P < 0.05 (versus control). (b) Mice in model and NNAV-treated group were immunized with 0.2 mL 5% SRBC on day 16 after treatment, and serum was collected on day 21. Mouse anti-SRBC antibody (Ab) levels were measured by haemolysis test. Values represent mean values ± SD for 10 mice per group. *P < 0.05 (versus model). (c) Isolated splenocytes from NNAV-treated or control mice were stimulated with LPS (10 *μ*g/mL) for 48 h and the proliferation rate of B cells was determined using CCK-8. Data represent mean values ± SD for 4 mice per group; triplicates were used for each mouse. *P < 0.05 (versus control).

NK cell activity could be used as the measure of nonspecific tumor killing capability of individuals. This function was expressed as percent lyses using LDH release from the target YAC-1 cells. As shown in Figure 1(a), NNAV at doses of 40 *μ*g/kg and 80 *μ*g/kg increased NK cell cytotoxicity after 21 days of treatment (*P* < 0.05, versus control).

Serum specific antibody test reflected the effects of NNAV on humoral immune response. The results are shown in Figure 1(b); in comparison with the normal control, the generation of mouse anti-SRBC antibody (Ab) in model group was significantly increased, which suggests that the sensitization caused by SRBC has been made successfully. NNAV at doses of 40 *μ*g/kg and 80 *μ*g/kg markedly increased antibody generation (*P* < 0.05, versus model). To test the effects of NNAV on B cells, the mitogen LPS stimulated B-cell proliferation was assessed. As shown in Figure 1(c), administration of NNAV greatly enhanced the ability of B-cell proliferation (*P* < 0.05, versus control).

**Figure 2:**
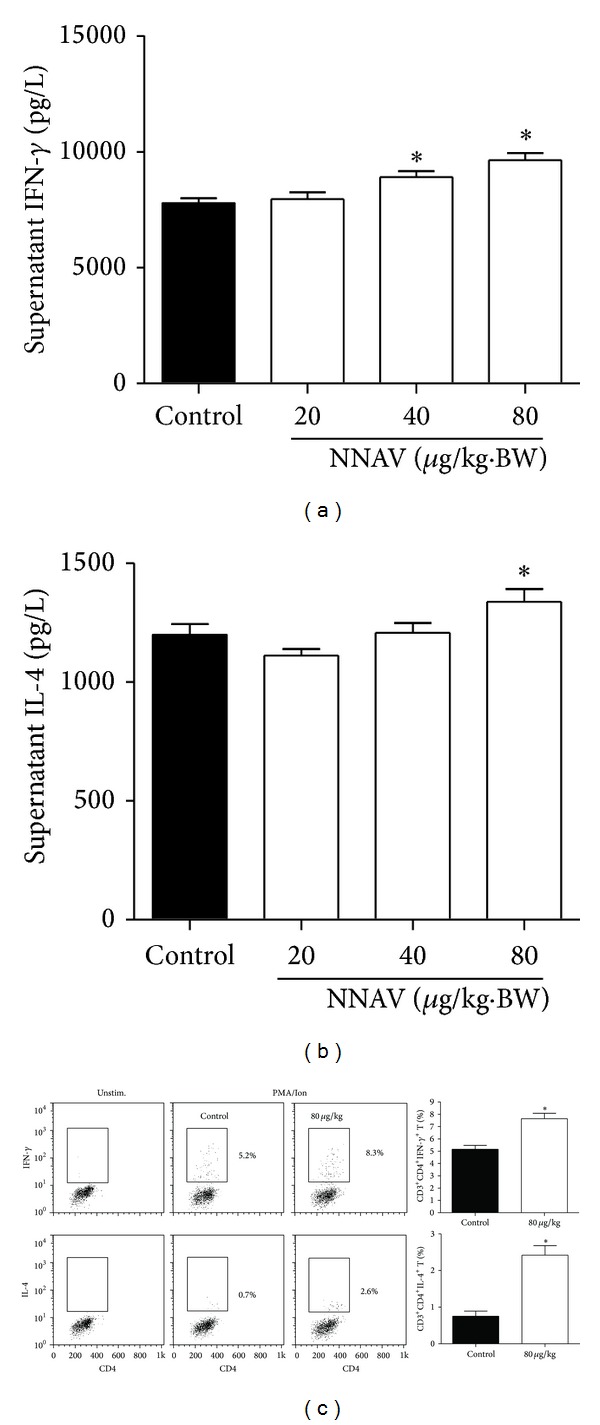
NNAV increased IFN-*γ* and IL-4 from Th1 and Th2 CD4 T cells. The NNAV solution was orally administered to ICR mice daily for 21 days, and control mice were given water. Splenocytes isolated from NNAV and control mice were stimulated with ConA (5 *μ*g/mL) for 48 h. The supernatant was collected for the determination of the concentration of IFN-*γ* (a) and IL-4 (b). Data represent mean values ± SD for 4 mice per group; triplicates were used for each mouse. *P < 0.05 (versus control). (c) For the determination of the expression of IFN-*γ* and IL-4 in CD3^+^CD4^+^T cells, splenocytes were collected and stimulated with PMA/Ion at the presence of BFA for 6 h. Data present mean values ± SD for 4 mice per group. *P < 0.05 (versus control).

**Figure 3:**
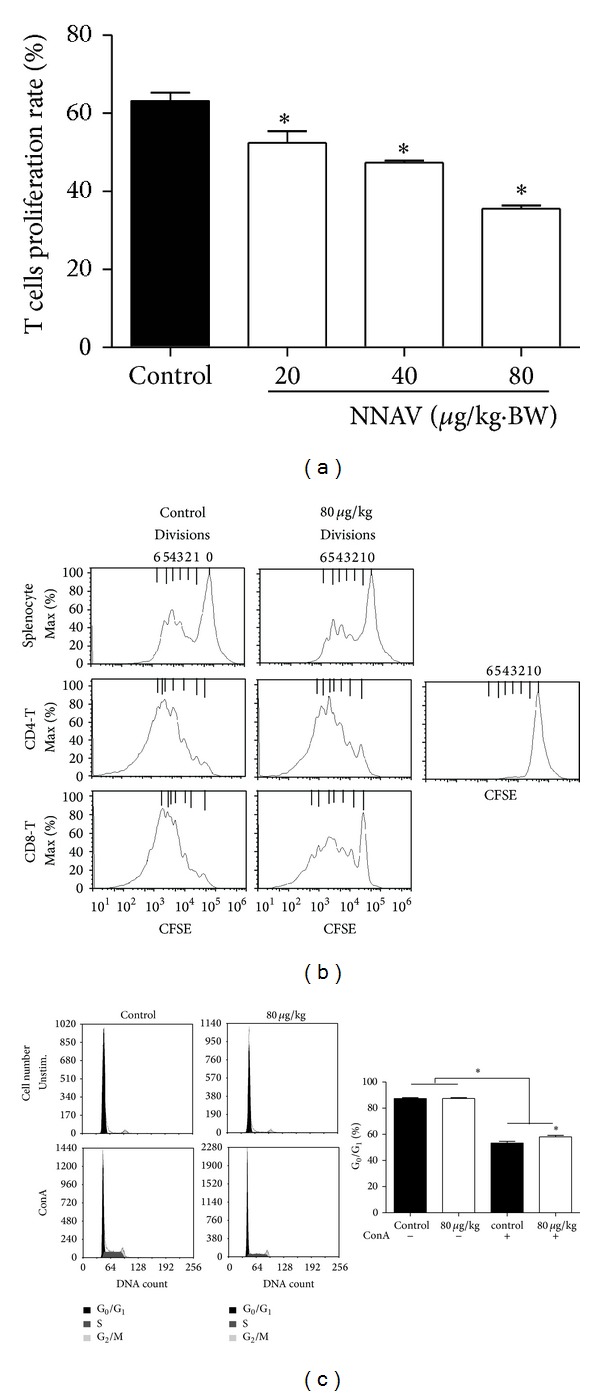
NNAV inhibited T-cell proliferation. After 21-day administration of NNAV or water, splenocytes were isolated from NNAV-treated or control ICR mice. (a) Splenocytes were stimulated with ConA (5 *μ*g/mL) for 48 h, and the proliferation rate of T cells was determined using CCK-8. Data present mean values ± SD for 4 mice per group; triplicates were used for each mouse. *P < 0.05 (versus control). (b) Splenocytes were stained with CFSE and stimulated with ConA (5 *μ*g/mL) for 72 h. The cell signals were obtained with or without gating on CD4^+^ or CD8^+^T subsets with flow cytometer. The cell division was represented by the histograms. This is representative of three experiments. (c) Splenocytes were stimulated with ConA (5 *μ*g/mL) for 48 h. Then cells were fixed with 70% ethanol and stained with PI for the cell cycle phase distribution analysis with flow cytometer. Data represent mean values ± SD for 6 mice per group. *P < 0.05 (versus control).

Th1 and Th2 cells, which mainly produce IFN-*γ* and IL-4, provide helper roles on innate and humoral immune responses [30]. As shown in Figure 3, NNAV markedly increased (*P* < 0.05, versus control) the secretion of IFN-*γ* (Figure 2(a)) and IL-4 (Figure 2(b)) and helped CD4 T-cell differentiate to Th1 (CD3^+^CD4^+^IFN-*γ* ^+^T) and Th2 (CD3^+^CD4^+^IL-4^+^T) subsets (Figure 2(c)).

### 3.2. NNAV Suppressed CD4 Th17 and CD8 Cellular Immune Activity

The effects of NNAV on splenocytes proliferation in response to T cell mitogen ConA are shown in Figure 3(a). All doses of NNAV showed a significant inhibition on cell proliferation (*P* < 0.05, versus control). For monitoring the effects of NNAV on T-cell division, splenocytes were stained with CFSE, and the retained CFSE label was distributed to each daughter cell when the cell divided. After stimulation with ConA for 72 h, the effects of NNAV on CD4 or CD8 T-cell division were analyzed by gating on CD4^+^ or CD8^+^ subsets of T cells. As shown in Figure 3(b), NNAV treatment (80 *μ*g/kg) demonstrated an inhibitory effect on ConA-induced splenocyte CD4 and CD8 T-cell division. Because NNAV inhibited T-cell proliferation and division, the influence of NNAV on cell cycle distribution was measured by staining PI. As presented in Figure 3(c), 53% of ConA-stimulated T cells were at G_0_/G_1_ phases and NNAV at 80 *μ*g/kg arrested 58% of the cells at G_0_/G_1_ phases. No sub-G_1_ peak in the cell cycle was observed after NNAV administration, indicating that no apoptosis was triggered. NNAV at the dose used also had no influence on splenocytes vitality (data not shown).

**Figure 4:**
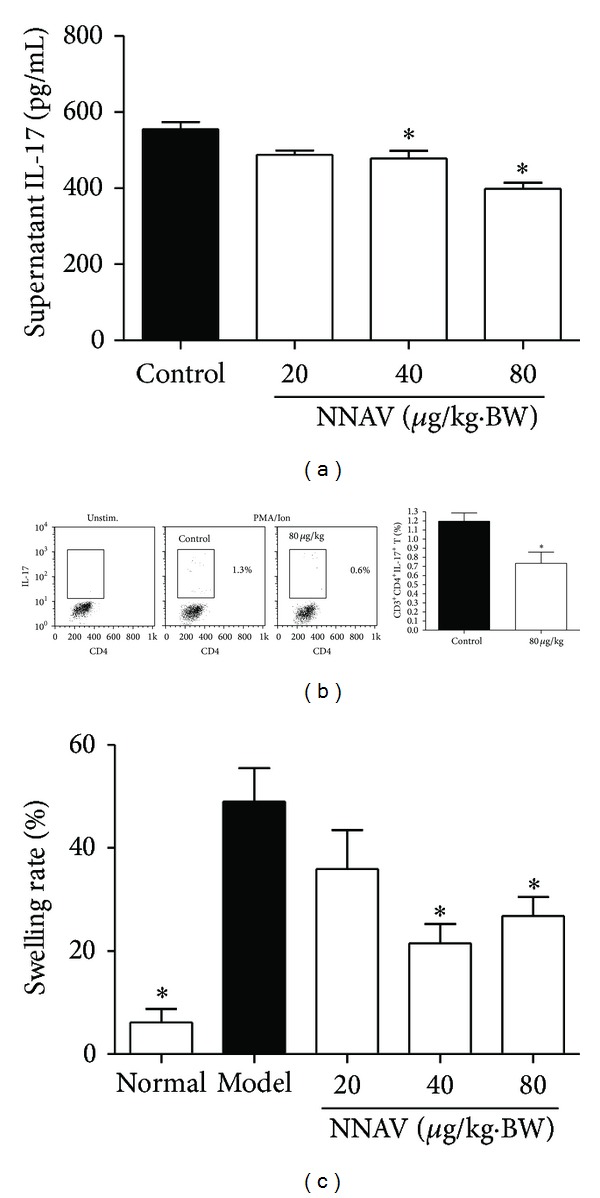
NNAV inhibited Th17 CD4 T-cell activity. The NNAV solution was orally administered to ICR mice for 21 days, and control mice were given water instead. (a) Splenocytes isolated from NNAV and control mice were stimulated with ConA (5 *μ*g/mL) for 48 h. The supernatant was collected for the determination of the concentration of IL-17. Data represent mean values ± SD for 4 mice per group; triplicates were used for each mouse. *P < 0.05 (versus control). (b) Splenocytes were stimulated with PMA/Ion at the presence of BFA for 6 h, and the expression of IL-17 in CD3^+^CD4^+^T cell was detected with flow cytometer. Values represent mean values ± SD from 4 mice per group. *P < 0.05 (versus control). (c) DTH response was determined in DNFB-immunized mice at 24 h after challenge by measuring ear swelling. Data represent mean values ± SD for 12 mice. *P < 0.05 (versus model).

Since NNAV increased the Th1 and Th2 subsets differentiation (Figure 2(c)), we next explored whether Th17 cells were involved in the inhibitory effects on CD4 T-cell. The results in Figures 4(a) and 4(b) showed a marked inhibition on IL-17 secretion and Th17 (CD3^+^CD4^+^IL-17^+^T) cell differentiation. IL-17 plays a capital role in delayed-type hypersensitivity (DTH) reaction [27]. We further measured the DTH response to DNFB; as shown in Figure 4(c), the ear swelling rate in the model group was significantly increased compared with normal group (*P* < 0.05), indicating that the DTH model in mice was made successfully. The ear swelling rate was significantly decreased in mice treated with NNAV at the doses of 40 *μ*g/kg and 80 *μ*g/kg compared with the model group (*P* < 0.05).

### 3.3. NNAV Partly Restored Dexamethasone-Induced Immunodepression

**Figure 5:**
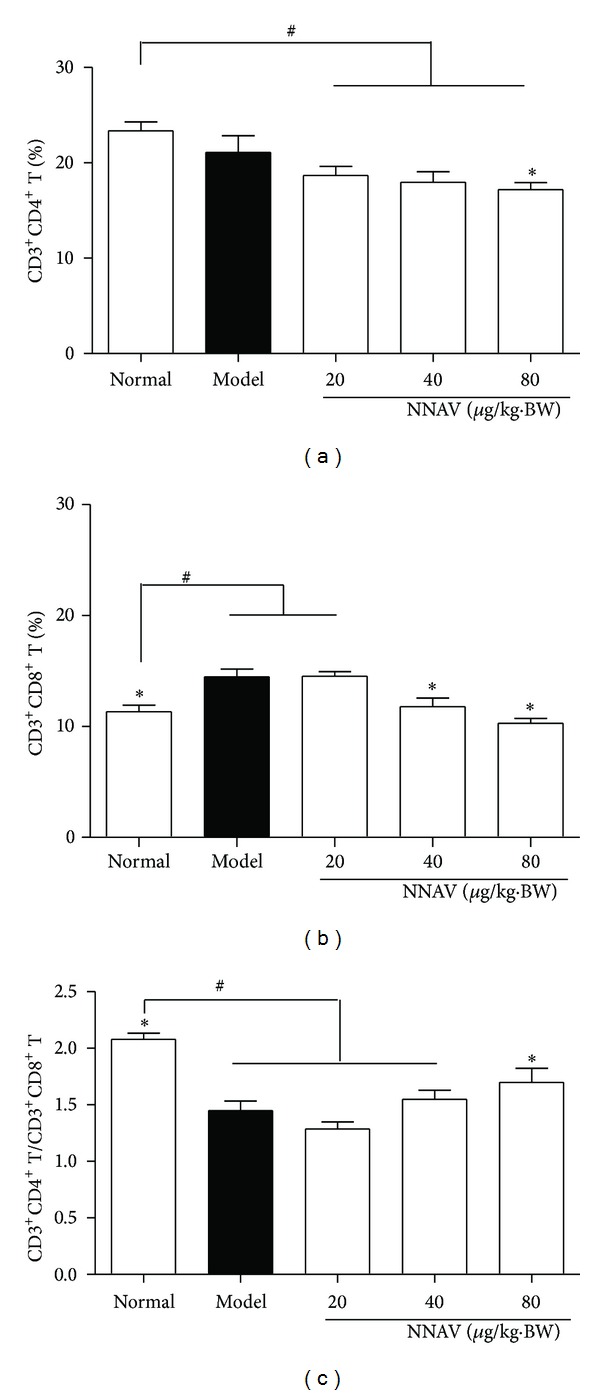
NNAV increased the CD4/CD8 T cell ratio in DEX-induced immunosuppressed mice. The DEX-induced immunosuppressed C57BL/6J mice were treated with NNAV for 21 days. Then splenocytes were prepared and stained with CD3e-PE-cy5, CD4-PE, and CD8a-FITC for analysis with flow cytometer. The percentage of CD3^+^CD4^+^T-cell (a) and CD3^+^CD8^+^T-cell (b) and the ratio of the two (c) are shown. Data represent mean values ± SD for 8 mice per group. *P < 0.05 (versus model); ^#^ *P* < 0.05 (versus normal).

Immunomodulatory study of NNAV was also performed using the DEX-induced suppressed mouse model. To study whether the changes of activities of T cells by DEX were altered by NNAV, T-cell subset analysis was conducted with flow cytometer. As shown in Figure 5(a), the decrease in CD4 T-cell subset (CD3^+^CD4^+^T) in the model group was further decreased when NNAV was given, especially at the dose of 80 *μ*g/kg (*P* < 0.05, versus normal). As shown in Figure 5(b), DEX alone could significantly increase the percentage of CD8 T-cell subset (CD3^+^CD8^+^T) compared with the normal group (*P* < 0.05); when combined with NNAV treatment, the percentage was markedly decreased (*P* < 0.05, versus model). The ratio of CD4 T-cell subset to CD8 T-cell subset (CD3^+^CD4^+^T/CD3^+^CD4^+^T) was significantly decreased in model group (*P* < 0.05, versus normal). Although NNAV decreased both CD4 and CD8 T cells, the decrease in CD8 T cells was much more robust than that in CD4 T cells, and thus the ratio of CD4/CD8 was increased (Figure 5(c)).

**Figure 6:**
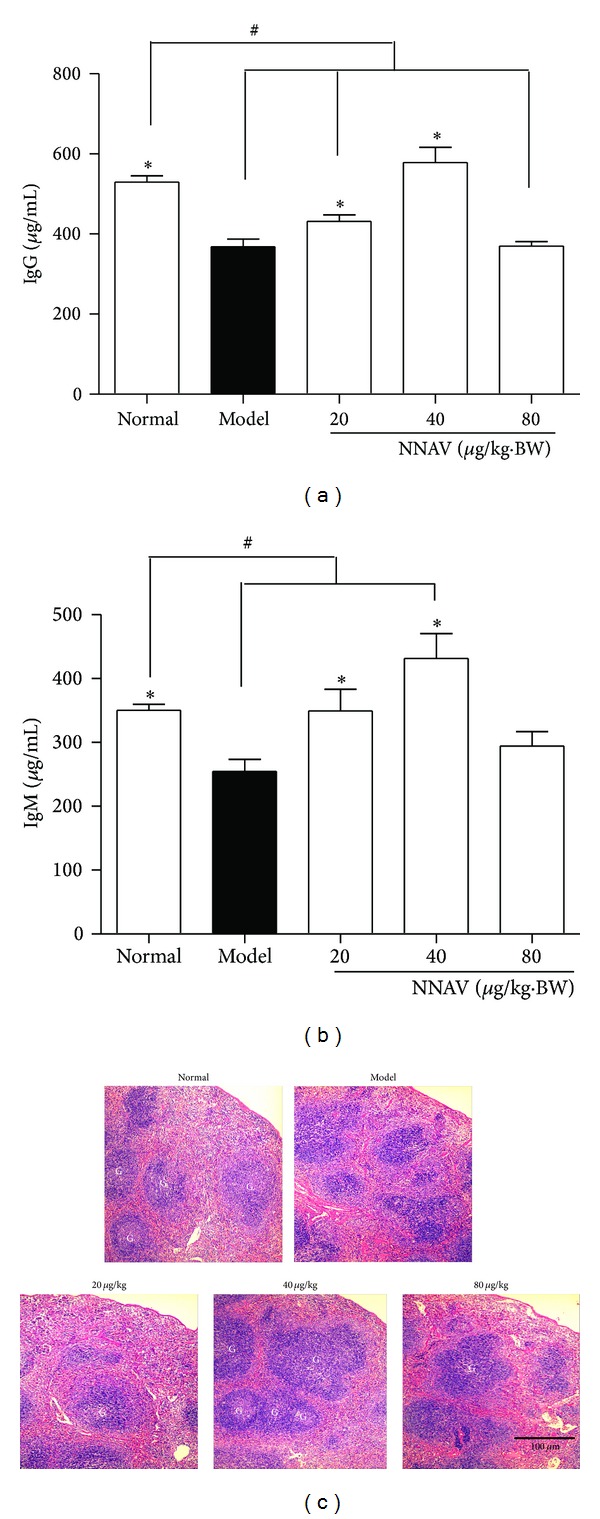
NNAV restored humoral immune responses in DEX-induced immunosuppressed mice. The DEX-induced immunosuppressed C57BL/6J mice were treated with NNAV or water for 21 days. Blood samples were collected and serum was prepared for the determination of IgG (a) and IgM (b) with ELISA. Data represent mean values ± SD for 8 mice per group. *P < 0.05 (versus model); ^#^ *P* < 0.05 (versus normal). (c) Spleen sections were stained with hematoxylin and eosin and examined with a microscope at 100x magnification. G: germinal center (light staining center). Scale bar: 100 *μ*m.

The effects of NNAV on serum immunoglobulin in immune suppressed mice were determined by measuring the two major components, IgG and IgM concentrations. Compared with normal group, IgG (Figure 6(a)) and IgM (Figure 6(b)) were significantly decreased in model group (*P* < 0.05). However, these decreases were significantly reduced by the combined administration of NNAV, particularly at the dose of 40 *μ*g/kg (*P* < 0.05 versus model). In spleen, germinal centers (GCs) play an important role in T-cell dependent, antigen-induced humoral immune response. As shown in Figure 6(c), DEX inhibited the formation of GCs. When mice were treatment with NNAV, the areas of GCs were recovered particularly at the dose of 40 *μ*g/kg.

## 4. Discussion

The present study conducted the first in vivo evaluation of NNAV on three major components of the immune response. The main findings are the following: NNAV could enhance the innate and adaptive humoral immune responses while inhibiting cell-mediated immune responses. NNAV could partly reverse the immune suppression induced by long-term DEX treatment. These results lay the foundation that NNAV could be a valuable agent for the treatment of a number of autoimmune diseases.

NK cells are a major component of the innate immune system, and they can recognize pathogen-infected and tumor cells without antibody and MHC [31]. The LDH release assay used in our study was widely used to examine the basal activity of NK cells [32, 33]. NK cells together with other cells, such as T cells and mononuclear phagocytes, produce multiple cytokines to mediate and regulate the function of NK cells. And IFN-*γ* is the most important cytokine in regulating NK cell activity [34]. Thus, in our study, the NNAV-induced enhancement of NK cell activity (Figure 1(a)) may be attributed to the direct or indirect effect of the stimulatory effect of NNAV on IFN-*γ* production (Figure 2(a)).

The primary antibody response to the T-dependent antigen SRBC was reported to be a sensitive endpoint to assess drug-induced alteration of the humoral immunity [35]. The production of the anti-SRBC antibody is closely associated with the cooperation and interaction with antigen presenting cells (APCs), T helper (Th) cells, and B cells [35]. The APCs uptake the antigen and present it to Th cells; then Th cells produce IL-4 to help B cells to differentiate and mature [36]. In the present study, the administration of NNAV improved the anti-SRBC antibody production (Figure 1(b)), reflecting the augmentation of humoral response to SRBC. This effect may be attributed to the stimulation of IL-4 production (Figure 2(b)) and B cell proliferation (Figure 1(c) by NNAV.

T cells have evolved to protect against intracellular infections and to help B cell respond to extracellular microbes [30, 37]. T cells not only play a helper role in the development of T-cell-mediated autoimmune disease but also have a direct role in tissue inflammation [30, 38]. T lymphocyte proliferation is a crucial event in the activation cascade of cellular immune response. We found that mitogen ConA-induced T cell proliferation was significantly suppressed by NNAV (Figure 3(a)). Consistent with this finding, data of CFSE-labeled cell division assay indicated that NNAV inhibited both CD4 and CD8 T cells division (Figure 3(b)). And the cell cycle analysis demonstrated that NNAV arrested T cell proliferation at the G0/G1 phases without inducing apoptosis (Figure 3(c)). The activation of NF-*κ*B is critical for T cell activation [10, 39]. When T cells are activated by mitogens, I*κ*B-*α* (the inhibitor of NF-*κ*B) is phosphorylated and degraded, leading to NF-*κ*B translocates to the nucleus [40]. Previous study has reported that oral administration of NNAV could inhibit the protein levels of P-IKK-*α*, recover I*κ*B-*α* levels, and prevent the translocation of NF-*κ*B to the nucleus in the rat model of nephropathy [18]. We suppose that the inhibition of T cell proliferation and division by NNAV may also involve in suppression of NF-*κ*B.

CD4 T cells also known as Th cells are mainly classified into four major lineages, Th1, Th2, Th17, and T regulatory (Treg) cells, based on their functions and their pattern of cytokine secretion [41]. It is interesting to know that NNAV produced an enhancing effect on innate and humoral immune responses partly due to its stimulatory effects on IFN-*γ* and IL-4 production by Th1 and Th2 cells (Figure 2). However, NNAV produced an inhibitory effect on CD4 T cells proliferation. To figure out which component of CD4 T cells was inhibited by NNAV, we invested the influence of NNAV on IL-17 production, which is a signature cytokine secreted by Th17 cells [41, 42]. Data showed that NNAV provided an inhibitory effect on the IL-17 production and Th17 differentiation (Figures 4(a) and 4(b)), and this result was consistent with the report that the presence of IFN-*γ* and IL-4 could inhibit the differentiation of Th17 cells [43]. DTH response is a cell-mediated immune response, and a recent study reported that Th17 cells rather than Th1 cells were crucial in the development of DTH response in certain cases [27, 44]. The suppressive effects of NNAV on DTH response (Figure 4(c)) also revealed the inhibitory effect on Th17 cell function. The Treg cells provide a nonselective suppressing effect on both innate and adaptive immune responses [45], and in our study, NNAV provided a discordance influence on immune response; thus, we conjecture that NNAV may have no or little influence on Treg cells.

Similar regulatory effects of NNAV were obtained in DEX-induced immunosuppressed mice. DEX is a member of glucocorticoid and has immunosuppressant property [46]. The results confirmed that NNAV could decrease the percentages of CD4 and CD8 T cells after DEX administration. Furthermore, the inhibitory effect of NNAV on CD8 T cells was more robust compared to CD4 T cells, resulting in the increase of the CD4/CD8 ratio (Figure 5). These results indicate that CD8 T cells were more sensitive to the inhibitory effects of NNAV. The present study showed that long-term exposure of DEX reduced antibodies production. However, NNAV could significantly restore IgG and IgM production (Figures 6(a) and 6(b)). GCs development relies on the activation of B cells by T-dependent antigen [47]. GCs play a critical role for B cell generation and secretion of high affinity antibodies [47, 48]. In this study, we found that NNAV could inverse the suppressive effects of DEX on the GCs generation (Figure 6(c)). These results showed that although NNAV reduced the amount of CD4 T cells, the humoral immune response was still strengthened, which indicated that NNAV could produce a selectively inhibitory effect on few parts of CD4 T cell subtypes.

Recent evidence indicates that CD4 Th17 and CD8 T cells can contribute to tissue damage in some inflammatory and autoimmune diseases and could be targets for therapeutic intervention in some autoimmune diseases [38, 49–51]. Our current research indicates that NNAV could selectively inhibit CD4 Th17 and CD8 T cells.

In this study, the dose effect relationship of NNAV varied significantly. The exact reasons for this phenomenon are unclear. We speculate that NNAV is composed of many active components and each of them produces different effects on immune system. This may result in complex dose effect relationship in different physiological indexes.

## 5. Summaries 

The present study documented that NNAV could strengthen the innate and humoral immune responses through the augmentation of NK and B cell function, or indirectly through the improvement on Th1 and Th2 cytokines secretion. On the other hand, NNAV could inhibit CD4 Th17 and CD8 T cell function. NNAV also partly improved the immune functions of DEX-induced immune depressed mice. During the study period, no death or any sign of clinical manifestation of toxicity was observed in mice administered NNAV at the doses of 20, 40, and 80 *μ*g/kg (Table 1). These findings suggest the therapeutic potential of NNAV on the treatment of autoimmune diseases.

## Figures and Tables

**Table 1:** NNAV had no effect on body weight, lymphoid organ index, or T lymphocyte subpopulations in normal mice.

	Control	20 *μ*g/kg	40 *μ*g/kg	80 *μ*g/kg
Body weight	34.41 ± 1.95	35.24 ± 2.19	33.07 ± 2.51	35.61 ± 1.15
Spleen index	38.17 ± 3.21	44.17 ± 4.18	40.55 ± 9.02	38.17 ± 6.58
Thymus index	9.83 ± 2.46	11.65 ± 2.95	11.52 ± 3.93	9.99 ± 3.96
CD4-T %	25.00 ± 1.99	23.59 ± 6.25	26.06 ± 3.47	25.86 ± 4.10
CD8-T %	8.46 ± 2.33	7.74 ± 1.38	7.82 ± 1.37	7.51 ± 1.89
CD4-T/CD8-T	3.09 ± 0.60	3.31 ± 0.33	3.27 ± 0.55	3.36 ± 0.44

After 21 days orally given NNAV or water, all ICR mice were sacrificed; spleen and thymus of each mouse were collected and weighted immediately. The T-lymphocyte subpopulations in spleen were measured by flow cytometer by gating on CD3^+^CD4^+^ (CD4-T) or CD3^+^CD8^+^ (CD8-T) cell population. The data present means ± SD; *n* = 8.
